# Drug reprofiling history and potential therapies against Parkinson’s disease

**DOI:** 10.3389/fphar.2022.1028356

**Published:** 2022-10-26

**Authors:** Komal Latif, Aman Ullah, Anastasiia D. Shkodina, Dmytro I. Boiko, Zakia Rafique, Badrah S. Alghamdi, Mohamed A. Alfaleh, Ghulam Md. Ashraf

**Affiliations:** ^1^ Riphah Institute of Pharmaceutical Sciences, Riphah International University, Islamabad, Pakistan; ^2^ Shifa College of Pharmaceutical Sciences, Shifa Tameer-e-Millet University, Islamabad, Pakistan; ^3^ Department of Neurological Diseases, Poltava State Medical University, Poltava, Ukraine; ^4^ Municipal Enterprise “1 City Clinical Hospital of Poltava City Council”, Poltava, Ukraine; ^5^ Department of Psychiatry, Narcology and Medical Psychology, Poltava State Medical University, Poltava, Ukraine; ^6^ Neuroscience Unit, Faculty of Medicine, King Abdulaziz University, Jeddah, Saudi Arabia; ^7^ King Fahd Center for Medical Research, King Abdulaziz University, Jeddah, Saudi Arabia; ^8^ Faculty of Pharmacy, King Abdulaziz University, Jeddah, Saudi Arabia; ^9^ Division of Vaccines and Immunotherapy, King Fahd Center for Medical Research, King Abdulaziz University, Jeddah, Saudi Arabia; ^10^ Department of Medical Laboratory Sciences, College of Health Sciences, University of Sharjah, Sharjah, United Arab Emirates

**Keywords:** central nervous system, drug repurposing, Parkinson’s disease, drug discovery, neurodegeneration

## Abstract

Given the high whittling down rates, high costs, and moderate pace of new medication, revelation, and improvement, repurposing “old” drugs to treat typical and uncommon illnesses is progressively becoming an appealing proposition. Drug repurposing is the way toward utilizing existing medications in treating diseases other than the purposes they were initially designed for. Faced with scientific and economic challenges, the prospect of discovering new medication indications is enticing to the pharmaceutical sector. Medication repurposing can be used at various stages of drug development, although it has shown to be most promising when the drug has previously been tested for safety. We describe strategies of drug repurposing for Parkinson’s disease, which is a neurodegenerative condition that primarily affects dopaminergic neurons in the substantia nigra. We also discuss the obstacles faced by the repurposing community and suggest new approaches to solve these challenges so that medicine repurposing can reach its full potential.

## 1 Introduction

**FIGURE 1:**
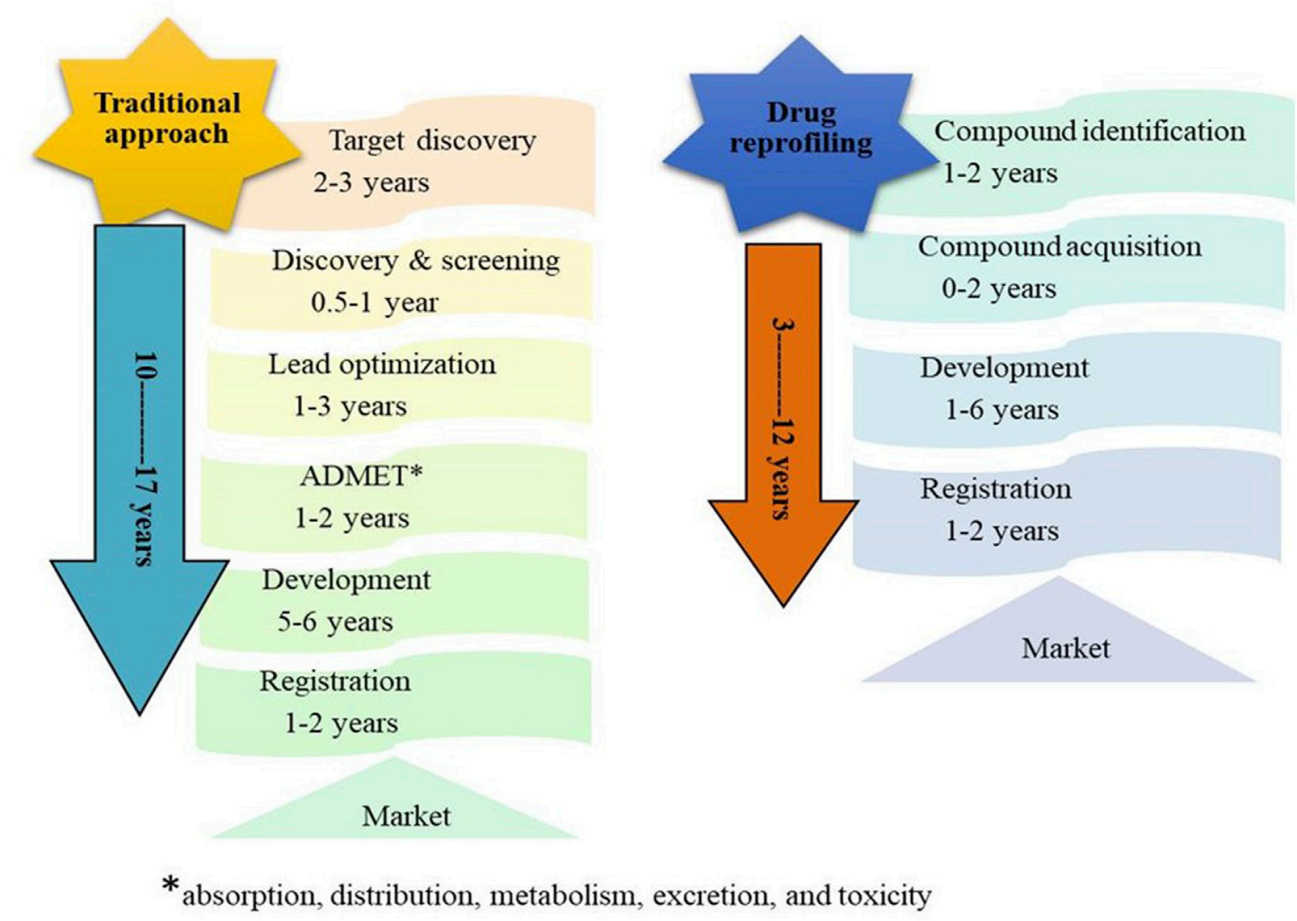
Comparison analysis between the traditional drug discovery approach and drug reprofiling.

Drug reprofiling history goes back to 1950; however, Ted T Ashburn and Karl B. Thar were the first to introduce the inception of drug repositioning in 2004 (Langedijk et al., 2015). Initially, people were unaware of this term, although this method was practiced in the late 1990s with the repositioning of thalidomide (Emanuel Almeida Moreira de Oliveira1, 2018). It is a fact that traditional drug development is complicated and tiresome (Gupta et al., 2021). Drug reprofiling or redirecting is a very attractive, economical, and time-saving process because this approach includes adding newer indications to the previously existing drugs (Steinhagen, 2011). This approach has succeeded in reducing the total period of drug development on average by 3–12 years, as shown in Figure 1. According to one of the studies in recent years, more than 30% of the US Food and Drug Administration (FDA)-approved drugs and vaccines have undergone the drug repurposing process (Jin and Wong, 2014). This tremendous achievement has opened the doors for researchers and drug developers interested in drug repurposing (Schonfeld, 2014).

Currently, many pharmaceutical firms are involved in drug research and development and are looking for innovative and economic approaches to treat diseases in better ways (Khanna, 2012). Such firms have allocated enormous proportions of money for research and development to support drug discovery and development. In recent years, it has been observed that research and development budgets have been significant (Mizushima, 2011). The massive success in repositioning sildenafil (Viagra), one of Pfizer’s products, has proved the landmark in drug repositioning (Dhir et al., 2020). The phase I clinical trial of sildenafil had a minimal effect against angina pectoris (primarily indication) with marked penile erection (Lue, 2000). Later, in 1998, researchers considered sildenafil to be the only regimen for erectile dysfunction and marketed it in the U.S under the brand name Viagra (Srinath and Kotwal, 1999).

Similarly, thalidomide was initially withdrawn from clinical use and was later rediscovered for its secondary action (Bartlett et al., 2004). Thalidomide was developed as a sedative and recommended to pregnant women to treat morning sickness, but this drug caused severe birth skeletal abnormalities in children (Vargesson, 2019). Thalidomide was banned due to its side effects, but later on, it was rediscovered as an inhibitor of TNF-α and was used to treat the condition erythema nodosum laprosum (ENL) (Ashburn and Thor, 2004a). It is also antiangiogenic, which led to its use as an anticancer agent for treating multiple myeloma (Gillies, 2016). Hence, there is always a possibility of repurposing and rediscovering a drug (Fetro and Scherman, 2020). Ramosetron is another drug that was initially used as an antiemetic (Desai et al., 2013). Later, it was reprofiled for irritable bowel syndrome because of its side effect, constipation (Graul et al., 2009). Therefore, drug repurposing includes scientific recreation of pharmacological activities of current drugs (Ashburn and Thor, 2004b).

Parkinson’s disease is a condition that still has a lot of unclear questions about its treatment despite a long history of its study (Seppi et al., 2019). More and more drugs that have a pharmacodynamic effect on the components of the pathogenesis of PD are undergoing clinical trials in order to optimize modern therapy and search for new ways to use known drugs (Deleu et al., 2002).

## 2 Significance of drug reprofiling

Due to its associated issues, the failure of traditional drug discovery has diverted the focus toward drug reprofiling (coined as drug repurposing, drug repositioning, drug re-tasking, or therapeutic switching), which is less time-consuming, cost-practical, and more effective. As the pharmacologist and Nobel laureate James Black said, “The most fruitful basis for the discovery of a new drug is to start with an old drug” (Pantziarka et al., 2018).

Reprofiling has an extra advantage over the traditional approach as new approaches overcome major drug discovery problems (Ashburn and Thor, 2004a). The survey report of expenses utilized in reprofiling in 2007 concluded that the cost to reprofile a drug averages $8.4 million (Agrawal, 2015). The success rate of reprofiled drugs is also higher than that of the traditional approach because of the established profiles of these compounds (Pushpakom et al., 2019). According to a survey report published in 2007, only 25% of drugs from phase II and 65% from phase III clinical trials reached the market compared to new molecular entities, which are 10% and 50% (Pantziarka et al., 2018). In addition, the complete picture of the successful report from the preclinical stage to the approved status is reported in Table 1 as drug reprofiling has additional significance over standard drugs because the repurposed drug already has a different test for various toxicity and side effects (Polamreddy and Gattu, 2019). These drugs have already passed through clinical trials, which reduces the development cost for prescriptions (Sun et al., 2016). According to a recent report based on a survey of 30 pharmaceutical industries and biotechnology companies, introducing a drug again as repurposed averages $8.4 million, while the price for research and development of a new 101 molecule is very high, averaging $41.3 million (Naylor et al., 2015). They also have a higher success rate than the original drugs because of known and tested information regarding their pharmacology, formulation stability, potential toxicity, safety, and adverse effects (Wen et al., 2015). However, introducing a new drug to the market requires clinical trials, scrutinizing tests on different models, and might be a waste of time, money, and effort (Califf, 2006). Instead of submitting a new drug to the market, using medications with known indications is more favorable (Rask-Andersen et al., 2011). Developing a new drug costs roughly one billion dollars, while reprofiling takes 60% less time than developing a novel drug and is less costly (Silverdale et al., 2005a; Silveira et al., 2019a).

**TABLE 1:** Success stories in drug repositioning.

S. no	Drug name	Primary indication	Primary manufacturer	Repositioning indication	Repositioning manufacturer	Year (FDA approval)/current status
1	Amitriptyline	Antidepressant	Sandoz	Neuropathic pain	Astra Zeneca	2005
2	Amphotericin B	Antifungal	------	Leishmaniasis	NeXstar Pharmaceuticals	1997
3	Aspirin	Analgesic/anti-inflammation	Many	Anti-platelet/stroke/heart attack	-------	------
4	Atomoxetine	Parkinson’s disease	Eli Lilly	Attention-deficit hyperactivity disorder (ADHD)	Eli Lilly	2002
5	Bupropion	Antidepressant	GSK	Smoking cessation	GSK	1997
6	Bleomycin	Antibiotic	BMS	Cancer	Kayaku/BMS	1973
7	Bromocriptine	Parkinson’s disease	Sandoz	Type II diabetes	Novartis	2009
8	Buprenorphine	Pain	Reckitt Benckiser	Opiate dependency	Reckitt Benckiser	2002
9	Chlorpromazine	Antiemetic/antihistamine	Rhone-Poulenc	Non-sedative tranquilizer	SmithKline	Not clear
10	Clofazimine	Tuberculosis	Novartis	Leprosy	Novartis	1986
11	Cyclosporine	Organ transplant rejection		Psoriasis/RA	Novartis	1997
12	Cycloserine	Tuberculosis		CNS		
				disorder	Many	Many
13	Dapoxetine	Analgesic/antidepressant	Eli Lilly	Premature ejaculation	Johnson & Johnson	2004
14	Duloxetine	Antidepressant/GAD	Eli Lilly	Stress urinary incontinence	Eli Lilly	2004
	------------	---------		Fibromyalgia	Eli Lilly	2008
				Musculoskeletal pain	Eli Lilly	2010
15	Donepezil	Alzheimer’s disease	Eisai	Dementia	Eisai/Pfizer	2006
16	Eflornithine	Cancer/				
		Anti-infective	Bristol-Myers Squibb	Hirsutism		
				Sleeping sickness	Gillette Aventis	1990
						2000
17	Etanercept	Rheumatoid arthritis	Pfizer	Plaque psoriasis	Amgen/Pfizer	2004
18	Fluoxetine	Antidepressant	Eli Lilly	Premenstrual dysphoria	Eli Lilly	2000
19	Finasteride	Hypertension	Merck	BPH	Merck	1992
				Male		
				pattern	Merck	1997
				baldness		
20	Galantamine	Polio/paralysis/anesthesia	Sopharma	Alzheimer’s disease	Many	2001
21	Gabapentin	Seizure	Parke-Davis	Post herpetic neuralgia	Parke-Davis	2004
22	Glycopyrronium	Anti-ulcer	Sosei/	COPD	Sosei/Novartis	2015
			Novartis			
				Excessive underarm		2018
				sweating		
			
23	Ibuprofen	Inflammation/pain	Boots laboratories	OA/RA/headache	-------	-------
				/migraine		
24	Imatinib	Chronic myelogenous leukemia	Novartis	Gastrointestinal stromal tumors	Novartis	2001
25	Infliximab	Autoimmune diseases	Janssen Biotech	Crohn’s disease	Janssen Biotech	1998
26	Mifepristone	Pregnancy termination	Danco Laboratories	Psychotic major depression	Corcept	2000
27	Minoxidil	Hypertension	Pharmacia &Upjohn	Hair loss	Pfizer	1998
28	Methotrexate	Cancer	-------	Psoriasis/RA	Barr Labs	2001
29	Naltrexone	Opioid/alcohol addiction	Endo Laboratories	Weight loss	Orexigen/Jakeda	2014
30	Paclitaxel	Cancer	National Cancer Institute	Restenosis	Angiotech/Boston Scientific	2004
31	Phentolamine	Hypertension	Novartis	Impaired vision	Ocularis Pharma	
32	Paroxetine	Antidepressant	GSK	Menopausal hot flashes	GSK	2013
33	Pertuzumab	Various cancers	Genetech	HER-2/breast cancer	Genetech	2013
34	Ropinirole	Hypertension	SmithKline Beecham	Parkinson’s disease	GSK	1997
				Restless leg syndrome		
					GSK	2005
35	Raloxifene	Osteoporosis	Eli Lilly	Breast cancer	Eli Lilly	2007
36	Retinoic acid	Acne	--------	Acute myeloid leukemia	Hoffman-La	1995
					Roche	
37	Rituximab	Various cancers	Genetech/Biogen	Rheumatoid arthritis	IDEC	2004
38	Sibutramine	Antidepressant	Boots Company	Obesity	Abbott	1997
39	Sildenafil	Angina	Pfizer	Erectile dysfunction	Pfizer	1998
40	Sunitinib	GIST/RCC	Pfizer	Pancreatic tumors	Pfizer	2010
41	Thalidomide	Anti-nausea	Chemie Grüenthal	Leprosy	Celgene	1998
				Multiple myeloma		
						2006
42	Tadalafil	Anti-inflammatory/CV diseases	GSK	Male erectile dysfunction	Eli Lilly and ICOS	2003
43	Topiramate	Epilepsy	J&J	Obesity	J&J	2003

Companies like Pfizer, Novartis, Eli Lilly, Biovista, and SOM Biotech are involved in the drug reprofiling process (Sekhon, 2013). Due to its associated issues, traditional drug discovery failures have shifted the focus toward drug reprofiling, which is not a cost-effective, time-saving, and more effective technique (Parvathaneni et al., 2019). According to the pharmacologist and Nobel laureate James Black, “The most fruitful basis for discovering a new drug is to start with an old drug” (Pantziarka et al., 2018).

Drug re-profiling has an extra advantage compared to the traditional approach because the new methods overcome the significant problems of drug discovery (Ashburn and Thor, 2004a). According to a survey report in 2007, the average expense in reprofiling was $8.4 million (Agrawal, 2015). The established profiles of these compounds achieved the success rate of reprofiled drugs higher than that of the traditional approach (Tobinick, 2009). The survey report published in 2007 showed that only 25% of drugs from phase II and 65% from phase III clinical trials reached the market compared to new molecular entities, which are 10% and 50% (Pantziarka et al., 2018). A complete picture of the success report from preclinical to clinical trials is reported in Table 2 (Polamreddy and Gattu, 2019).

**TABLE 2:** List of drugs being repurposed and in clinical trials for PD.

Drug	MOA	Original use	Proposed use	Comments
**Tetracycline (** Bortolanza et al., 2018)	Inhibits the initiation of translation by binding to the 30S subunit	Antibiotic	Antiapoptosis , anti-inflammation, and MMP inhibition in PD	Phase II clinical trial
				Multitarget antibiotic
**N-Acetylcysteine (** Delgobo et al., 2019)	Mucolytic agent	For cystic fibrosis and acetaminophen toxicity	Antioxidant, anti-inflammatory agent, and neurotrophic factor	IV NAC raised brain glutathione levels in clinical trials
**MSDC-0160 (** Athauda and Foltynie, 2018; Savitt and Jankovic, 2019)	Stimulates progenitor cells to differentiate into brown-like fat cells rather than white fat cells *in vivo*	Originally formulated for type 2 DM	Targets MPC modulating cellular function	Phase I, preclinical. The Cure Parkinson’s Trust (CPT) is working on it and finding new approaches
β2AR agonist salbutamol (Magistrelli and Comi, 2019)	Aids in relaxation of smooth muscle in the lungs by coupling to a stimulatory G protein of adenylyl cyclase	Respiratory diseases	Reduces *SNCA* expression and is an inhibitor of	Use of β2AR agonists is rapidly growing
			microglia activation	
Simvastatin (Tong et al., 2018)	A specific inhibitor of (HMG-CoA)	It lowers cholesterol in cardiovascular diseases	Inhibits NADPH oxidase/p38 activation and enhances the expression of antioxidant proteins	Phase II. Recent studies have showed the protective effect of statins, but they disappeared when they were adjusted for cholesterol
	reductase, the enzyme that catalyzes the conversion of HMG- CoA to mevalonate			
Deferiprone (Sun et al., 2018)	Forms complexes with iron	Fe chelating agent	Study was conducted on the effect of conservative Fe chelation with	Phase II
			30 mg/kg/day of deferiprone in PD	
Exenatide (Aaseth et al., 2018)	Glucagon-like peptide receptor stimulator	Increases insulin release and decreases glucagon release	Neuroprotective ability	Phase II
				A first drug which slows PD progression
Ursodeoxycholic acid (UDCA) (Bell et al.,2018)	Reduces elevated liver enzyme levels by facilitating bile flow through the liver and protecting liver cells	Secondary bile; it reduces cholesterol absorption and dissolves gallstones	Improves mitochondrial function and redistributes Drp1 in fibroblasts	
				Phase II
				Improves mitochondrial function and redistributes Drp1 in fibroblasts
Isradipine (Liss and Striessnig, 2019)	Inhibits Ca^2+^ entry into excitable cells	L-type Ca^2+^ channel blocker	Neuroprotective ability	Phase III
				Final study results were expected in winter 2019
Ambroxol (Silveira et al., 2019b)	Stimulates mucus production and stimulates synthesis of the surfactant and their release by type 2 pneumocytes	Mucolytic agent	Increases levels of the GCase enzyme	Phase II
				Clinical trials may have a large impact on disease-modifying therapies in PD
Minocycline (Cankaya et al., 2019)	Suppresses viral replication by reducing T-cell activation	Antibiotic	Treatment induces functional regeneration that is dopaminergic neuron activity–dependent	Neuroprotective effects in PD experimental models have been reported since 2001
Doxycycline (Santa-Cecilia et al., 2019)	Inhibits bacterial protein synthesis by binding to the 30S ribosomal subunit	Antibiotic	Antiapoptotic and anti-inflammatory mechanisms	DOX
			involving the downregulation of MMPs	Inhibits α-synuclein aggregation and prevents cytotoxicity in dopaminergic cell lines
			
Atomoxetine (Yssel et al., 2018)	Prevents the reuptake of norepinephrine and inhibits the reuptake of dopamine	1 Noradrenaline reuptake inhibitor	It, alone or in combination, reduces the motor deficit induced by a nigrostriatal lesion in rats	Phase IV
		Treatment of ADHD		
**Omega-3 fatty acids**	Essential fatty acid of the diet, present in the brain	Essential fatty acid the of diet, present in the brain	Prevention of cognitive dysfunctions	Normalizing the antioxidant mechanism in the brain
(da Silva et al., 2008; Alquraan et al., 2019)				
**Topiramate (** Silverdale et al., 2005b)	Blocks voltage-dependent sodium and calcium channels	Epilepsy	Reduces levodopa-induced dyskinesia and manages impulse control disorder in PD	Terminated in Phase II
**Astemizole (** Styczynska-Soczka et al., 2017)	Competitive antagonism of histamine binding to cellular receptors	Second-generation H1 histamine antagonist	Improvement of motor functions and the survival rate	Withdrawal from the market due to rare fetal side effects
**Sex steroids (** Bourque et al., 2019)	Stimulate estrogenic actions in tissues such as the liver, bone, and cardiovascular system but known to block estrogen action	Selective estrogen receptor		
		modulators	Useful in erectile dysfunction related to PD	In order to develop personalized medicine, estrogens could be used in priority for women
**Rivastigmine (** Smith and Peall, 2018)	Inhibits both butyrylcholinesterase and acetylcholinesterase	Parasympathomimetic agent	Improves L-dopa availability and has favorable effects on cognition, psychiatric symptoms, and dementia	Phase II
				Guidelines from the American
				Academy of Neurology have
				recommended rivastigmine for
				patients with PD
**Efilevodopa (** Rao and Rao, 2009; Ogawa, 1994a; Ogawa, 1994b)	Delivers dopamine to the brain	Levodopa ethyl ester dopamine agonist	Increases the efficacy of levodopa	Phase III
**Istradefylline (KW-6002)** (Nagayama et al.,2019)	Exhibits inhibitory function on most of the tissues	Adenosine A2 receptor antagonist	To treat mood disorders in PD	Phase III
**NeuroCell** (Palmer et al.,2019)	Surgery	Cell transplant therapy	Surgery	Phase III

## 3 History and molecular pathogenesis of PD

PD is the second most known neurodegenerative disease (Seppi et al., 2019). Approximately 7–10 million humans around the globe are affected by this disease, i.e., approximately one percent of the world population (Ismail et al., 2019). In North America, 0.075 million newly diagnosed individuals are added up each year to this count (Crippa et al., 2019). James Parkinson, in 1817, published an essay, “Shaking Palsy” (Pandey, 2012). Later, William Rutherford Sanders, in 1876, was the first to use the term “Parkinson” in the medical panorama (Lewis et al., 2020).

Bradykinesia is the principal feature of PD along with other motor deficits, i.e., rest tremors, gait, postural instabilities, agitation, swallowing disturbances, and slurred speech (Pandey, 2012). Non-motor co-morbidities include cognitive disorders, neuropsychological disorders, sleep disorders, orthostatic hypotension, constipation, bladder dysfunction, and sexual dysfunction (Poewe and Mahlknecht, 2009; Shkodina et al., 2022). The central issue of currently available treatment is motor response fluctuation or on–off treatment (Emanuel and Karen, 2018). Another problem encountered after a few years of treatment was patients complaining of the wear-off effect (Pandey, 2012). The exact etiology of PD remains a challenge for researchers as about 85% of idiopathic PD and only 15% are caused by a mutation in specific genes responsible for altering functions of various proteins (Kalinderi et al., 2016). The proposed etiologies are thought to arise in genetically sensitive individuals or might have environmental impacts on the molecular levels, such as insecticides, other toxins, or teratogenic causes (Emanuel and Karen, 2018). Rotenone, an insecticide and toxin MPTP, was used to induce PD in animal studies (Emanuel and Karen, 2018). PD is a neurodegenerative disorder of aging individuals with predominantly slow degradation of dopaminergic neurons in the substantia nigra pars compacta part of the brain (involved in motor function), which subsequently results in a decline in levels of the neurotransmitter dopamine in the striatum (Maiti et al., 2017). Synuclein (Lewy bodies) aggregation in the brain is the hallmark of PD isolated in 1997 (Baba et al., 1998). α-Synuclein is an essential protein, and its aggregation results in motor deficits (George and Brundin, 2015). Its post-translational modification, such as oligomerization or false synuclein aggregation, causes PD (Stoker et al., 2018). Molecular alteration and underlying causes of PD are evaluated in different studies (Zhou et al., 2008). Protein kinases and signaling pathways that are linked, tested, and assessed for relation in PD are phosphatase and tensin homolog (PTEN)-induced putative kinase 1 (PINK1) and leucine-rich repeat kinase 2 (LRRK2) (Alessi, Sammler, 2022). PINK1 and LRRK2 with associated protein kinase B (AKT) and c-Jun N-terminal kinase (JNK) signaling pathways have proven to be strong footings in PD (Mehdi et al., 2016). α-Synuclein (SNCA) proteins are produced by soma cells and play a prime role in the pathophysiology of PD (Stefanis, 2012). Usually, α-synuclein is distributed in the axon and stays in nerve terminals (Uchihara and Giasson, 2016). They function as the maintenance of synaptic balance and transmission of nerve impulses (Bendor et al., 2013). Synuclein is a protein with three domains: the (amino) N-terminal domain, hydrophobic domain, and (carboxyl) C-terminal domain (Jagannatha Rao, 2007). The hydrophobic domain, also known as NAC, is essential for the conversion of synuclein to an oligomer; in addition, it is believed to mediate a conformational change to the random coil to the beta-sheet structure upon aggregation (Uversky and Eliezer, 2009). The presence of the NAC region in α-synuclein discriminates it from beta- and gamma synuclein, and it is responsible for the induction of accumulation of these proteins (Brás and Outeiro, 2021).

Cellular homeostasis involves protein degradation through the ubiquitin–proteasomal system (UPS) and different types of autophagy (Tolosa, 2010). Chaperone-mediated autophagy (LeWitt et al., 2007) pathways are involved in α-synuclein elimination under normal conditions (Tolosa, 2010). The SNCA sequence at the 95–99 residue VKKDQ configuration resembles the lysosomal surface receptor LAMP-2A (Tolosa, 2010). However, due to mutation in α-synuclein, binding and autophagy through lysosomes are disturbed, and they begin to oligomerize and aggregate within neurons (Gorman, 2008). Accumulation of α-synuclein in a considerable amount results in Lewy bodies, and neurons gradually become less functional and disappear as in PD pathogenesis, the neuron count in the substantia nigra is decreased (Fahn, 2003). Mutations in *LRRK2* genes also play a significant role in PD pathogeneses (Rocha et al., 2022). It has domains like protein kinase and GTPase, the later environment being dominant in pathological changes (Taylor and Alessi, 2020). Phosphorylation of a group of RAB proteins by LRRK2 causes radical changes in essential aspects of autophagy and lysosomal physiology (Alessi and Sammler, 2022). LRRK2 mutations encompass almost all PD categories, like familial PD, idiopathic late-onset PD, autosomal dominantly inherited PD, and sporadic PD (Mehdi et al., 2016).

The second most typical cause of falling recessive PD is an alteration in PTEN-induced PINK1, commonly termed DJ-1 (Balestrino and Schapira, 2020). It is responsible for handling mitochondrial DNA levels, ATP production, calcium handling, and regulating free radical generation, and alteration in these functions can lead to apoptosis (Schapira, 2008). This change in PINK1 causes a reduction in the kinase activity related to atypical PD and causes the early age onset and slow progression of the disease (Valente et al., 2004). Alteration in PINK1 functionalities is also linked to familial juvenile PD around 1–8% (Myhre et al., 2008).

One of the molecular pathways of PD pathogenesis is oxidative stress, which is caused by the accumulation of reactive oxygen species (ROS) because of a deficiency in antioxidant systems that leads to cell death, including apoptosis, parthanatos, necroptosis, and autophagic cell death (Trist et al., 2019). Some genetic risk factors are also associated with mitochondrial dysfunction in dopaminergic neurons, which makes a significant contribution to the development of oxidative stress in PD (Dias et al., 2013). This complexity and multidimensionality of the pathogenesis of PD make it difficult to find an appropriate drug therapy (Krüger et al., 2017).

## 4 Available treatments and their limitations

There are currently no disease-modifying treatments for PD, and dopaminergic medications constitute the mainstay of treatment (Stoker et al., 2018). Preparations of levodopa, the precursor of dopamine, are the most widely utilized, and they are given in combination with a dopa-decarboxylase inhibitor to reduce some of the side effects, such as nausea (Deleu et al., 2002). Ropinirole and rotigotine, which are dopamine agonists, are also used (LeWitt et al., 2007). Endogenous dopamine metabolism can be slowed using monoamine oxidase B inhibitors like rasagiline and selegiline, as well as catechol-O methyltransferase (COMT) inhibitors like entacapone (Chen and Swope, 2007). Treatments for PD can restore dopaminergic function in the striatum, resulting in improvements in motor symptoms (Calabresi et al., 2000). They do not, however, cure many non-motor symptoms, which are very disabling for many individuals (Pfeiffer, 2016). Some non-motor symptoms, such as postural hypotension and neuropsychiatric issues, may be exacerbated by therapy in a few cases (Worth, 2013).

The majority of people who receive dopamine replacement medication suffer aberrant involuntary movements, such as L-DOPA-induced dyskinesia (Myhre et al., 2008). It is debilitating, and there is only one drug that can help, amantadine (Buck and Ferger, 2010). Repurposing compounds that have been shown to be safe in humans at phase II or higher can be a very efficient way to get new therapies to patients quickly (Schein, 2020). Repurposing avoids many high-risk phases of the drug development process (Rudrapal et al., 2020). During repurposing, development for a further indication at phase IIa is significantly less costly, takes as little as 4 years, and has an ∼3,000 times higher chance of reaching patients than a novel drug (Singh et al., 2020). We focus on historical and modern techniques to discover possible repurposed medications, propose mechanisms to prioritize the testing of new compounds, and highlight hurdles, particularly in the translation from preclinical testing to phase II clinical proof-of-concept studies (Crippa et al., 2019).

Antidyskinetic effects of NMDA antagonists were described in animal models of PD, including the MPTP-lesioned non-human primate, around 30 years after the discovery of amantadine (NHP) (Jakowec and Petzinger, 2004). These findings prompted a re-evaluation of amantadine’s effects in PD, and two separate groups reported a reduction in L-DOPA-induced dyskinesia in patients on amantadine in 1998, arguing for the drug’s usage as an antidyskinetic agent (Myhre et al., 2008). The off-label use of immediate-release amantadine has been shown to provide significant relief of LID in up to one-third of patients (Vijverman and Fox, 2014). In some individuals, long-term amantadine use, at least, to date, in the immediate release form may be compromised by tachyphylaxis, which has been reported to occur as early as 6 months of usage (Deleu et al., 2002). Long-term use does, however, provide clinical benefits for many patients (Wolf et al., 2010). Amantadine is also poorly tolerated because of cognitive issues, like confusion and hallucinations, and some non-cognitive side effects, like ankle edema (Jackson et al., 2009). It is not appropriate for individuals with renal failure (deVries et al., 2019). Relatively better tolerability of the extended-release amantadine is observed by the once-daily dosing at night, but long-term clinical use is yet required to confirm this proposition (Sharma et al., 2018).

With a better understanding of the CB1 cannabinoid receptor’s role in the control of basal ganglia transmission, another possible repurposing candidate was identified in the mid-1990s (Johnston et al., 2019). Indeed, the CB1 agonist nabilone, which is used to treat chemotherapy-related nausea, was demonstrated to diminish LID in NHPs that had been lesioned with 1-methyl-4-phenyl-1,2,3,6-tetrahydropyridine (MPTP) (Johnston et al., 2019). These findings, however, have not led to widespread usage of nabilone in LID due to non-efficacy concerns (Johnston et al., 2019). It was hypothesized that focusing on it would alter firing patterns and lower LID in a way that had previously been validated, albeit more invasively, with deep-brain stimulation (Heumann et al., 2014). As a result of this idea, the anticonvulsant levetiracetam was identified as a potential repurposing candidate (Bezard et al., 2004). In the MPTP-NHP model, levetiracetam activates SV2A and exhibits strong antidyskinetic efficacy (Johnston et al., 2019). However, because the drug was poorly tolerated in the PD patient population, these improvements could not be converted into effectiveness in phase II trials (Wong et al., 2011). Nabilone and levetiracetam are two examples of repurposing drugs that emphasize the relevance of efficacy and tolerability (Crippa et al., 2019).

Exenatide, a well-known diabetic medication for type 2 diabetes and a glucagon-like peptide-1 (GLP-1) agonist, and nilotinib, a tyrosine kinase inhibitor, have both recently been repurposed and tested in PD patients (Fletcher et al., 2021). At the same time, nilotinib is used to treat chronic myelogenous leukemia; thus, data on their safety and tolerability in patient populations already exist, which has aided their advancement through clinical studies, which have shown promising results (Athauda and Foltynie, 2018). In toxin-based mouse models of nigrostriatal degeneration, exenatide has been demonstrated to have neuroprotective and neurorestorative effects, enhancing motor function, behavior, learning, and memory (Athauda and Foltynie, 2015). Nilotinib has been shown to improve misfolded *α*-synuclein, making it a promising candidate for lowering SNCA levels *via* autophagy (Pagan et al., 2016). PD has been linked to higher levels of c-abl, which is thought to enhance the phosphorylation and aggregation of SNCA (Lindholm et al., 2016). Furthermore, an increase in the c-abl activity reduces the action of parkin, a key protein in mitochondrial biogenesis whose mutations cause familial PD (Brahmachari et al., 2017). Nilotinib has been shown to attenuate exogenously expressed SNCA levels in mice and reduce SNCA-induced nigral degeneration (Wong and Krainc, 2017). However, because there was no placebo group in this study and significant baseline differences between the two small groups, it was impossible to comment on any potential clinical benefits of the medicine (Espay et al., 2020). Despite the promising results of preclinical research and the fact that another trial (NILO-PD) is now underway in the United States, there is no convincing evidence of nilotinib’s efficacy in PD patients (Stoker et al., 2018).

## 5 FDA-approved repurposed drugs for PD

The central nervous system (CNS) is the most important and crucial area for drug repositioning due to its complicated pathophysiology, complex anatomy, and extra barriers that make it difficult to understand (Messick et al., 1985). So the exact mechanism of action of already established drugs for CNS disorders is not clearly understood (Gilroy et al., 2004). CNS is being researched continuously to understand receptor profiling and the mode of action of already developed and marketed drugs to address these problems (Anighoro et al., 2014). The prevalence rate of neurodegenerative disorders is much more in the world population (Chandra et al., 2006). Still, the drug discovery and development of these disorders is shallow and does not meet the needs of the people (Ekins et al., 2019). So to cope with this world’s worst dilemma, it is the need of the hour to discover new therapies (Ashburn and Thor, 2004a). Drug repositioning can address this issue by finding new drug therapies and better combinations of drugs for increasing efficacy and decreasing side effects (Sun et al., 2016). This review discusses the historical and current status of FDA-approved repositioned medicines for PD, focusing on new approaches to identify potential drugs that can be repurposed and identifying their mechanism of action. We know that PD is the second most prevalent neurodegenerative disorder (Seppi et al., 2019). According to the previous literature review, more than 6 million people are affected by PD worldwide (Nadim et al., 2020). There is an intense demand to find therapies that will prevent and slow the extension of this progressive and chronic condition, which significantly affects the patient’s quality of life (Athauda and Foltynie, 2018). The already established treatment regimens for PD had some direct side effects, so new agent development through repositioning is inevitable due to the ease of work, reduced cost, and evolution time (Nussbaum, 2002). FDA-approved repositioned drugs for PD are mentioned in Table 3.

**TABLE 3:** Repurposed drugs reported in the literature with FDA-approved status for the treatment of Parkinson’s disease.

Drug	MOA	Original use/brand name/originator firm	New use/brand name/ repositioner firm	FDA approval of repurposed drugs	MOA of the new target
Ropinirole hydrochloride (Weintraub et al., 2006)	-----	Hypertension	Parkinson’s disease	September 1997	D2 agonist
					Dopamine agonist
		-----	Requip		
		SmithKline Beecham	GlaxoSmithKline		
Amantadine (Rascol et al., 2021)	Anticholinergic agent	Influenza, antiviral	Parkinson disease dyskinesia	August 2017	NMDA receptor antagonist
	M2 protein inhibitor	Symmetrel	GOCOVRI		Dopamine release and reuptake inhibitor
		Endo Pharmaceuticals	Adamas Pharmaceuticals		
Rasagiline (DeMaagd and Philip, 2015b)	-------	In 1960, antidepressant and antihypertensive developed	Parkinson’s disease Azilect	May 17	MAO-B
		in the 1970s. Aspro Nicholas	Teva Pharmaceuticals	2006	Inhibitor
Pimavanseri n	Inverse agonist and antagonist	Antipsychotic agent	Hallucinations and	29 April 2016	Non-dopaminergic, selective serotonin inverse agonist (SSIA) for the treatment of psychosis associated with PD
	activity at serotonin 5-HT2A receptors		delusions associated		
			with PD		
			Nuplazid		
			Acacia Pharma		
Memantine (Hsu et al., 2018)	------	Influenza and Alzheimer’s disease	Parkinson’s disease cognitive deficit	------	It inhibits enzyme NMDA receptor
		Merz & Co.			

### 5.1 Ropinirole

Ropinirole hydrochloride is one of the several ergoline D-2 receptor agonists (Kaye and Nicholls, 2000). SmithKline Beecham first developed it for hypertension; then, it was repositioned by GSK and approved by the FDA in 1997 for early and later PD (Zesiewicz and Hauser, 1999). Dopamine agonist drugs act by mimicking levodopa in the brain and improve problems associated with levodopa use (Antonini and Tolosa, 2009). Levodopa is the principal drug used for PD treatment (Fiala et al., 2003). Although it is the most potent therapy, its side effects include dyskinesias (involuntary muscle movement) and “on–off” symptoms, which are troublesome in long-term use (Thanvi et al., 2007). Alternatives that delay or reduce exposure to levodopa have been explored to improve the patient’s quality of life and reduce the risk of side effects (Schapira, 2005). To address levodopa-induced dyskinesia, ropinirole was successively repositioned for PD (Nyholm, 2003). Nowadays, according to NICE guidelines, dopamine agonists and monoamine oxidase B (MAO-B) inhibitors may be used for the correction of motor deficits in case it does not impact the quality of life (Stocchi et al., 2015).

### 5.2 Amantadine

The FDA approved amantadine in October 1966 as a prophylactic agent against influenza (Douglas, 1982). The exact mechanism by which it exerts its antiviral activity is unknown. However, it is believed to prevent the release of viral nucleic acid into the host cell by inhibiting the M2 viral protein (Skehel et al., 1978). During the 2009 pandemic flu season, the Centers for Disease Control and Prevention (CDC) found flu samples 100% resistant to amantadine (Dapat et al., 2012). This drug was accidentally discovered to be reducing symptoms of PD in 1969 (Hubsher et al., 2012). Amantadine hydrochloride (the antidyskinetic agent) was repositioned by Adamas Pharmaceuticals and approved by the FDA for treating dyskinesia in PD patients receiving levodopa-based therapy (Sharma et al., 2018). In August 2017, the FDA had approved the first and only drug for treating dyskinesia in PD patients (Chen et al., 2020). Amantadine treats dyskinesia by blocking the NMDA receptor, thus decreasing the inactivation of dopamine and blocking presynaptic dopamine reuptake, and prolonging its adequate time (Schaeffer et al., 2014). These repurposed molecules have proven safe in humans and can be a highly efficient method of rapidly bringing new treatments to patients (O'Connor and Roth, 2005). Repurposing bypasses many high-risk phases of the drug development process (Shineman et al., 2014).

### 5.3 Rasagiline

In early 1970, Aspro Nicholas first invented and patented rasagiline for hypertension (Entzeroth and Ratty, 2017). But in mid-2006, while identifying a potential repurposing candidate in the case of PD, the MAO inhibitor role of rasagiline was discovered (Guay, 2006). Rasagiline was identified as an MAO-B inhibitor effective as monotherapy (Fiedorowicz and Swartz, 2004). MAO-B inhibitors can be prescribed as adjuvant therapy for motor symptoms, and it is supposed that they have a lower risk of hallucinations than dopamine agonists (Oertel and Schulz, 2016). Since the accumulation of SNCA aggregates leads to an increase in oxidative stress, mitochondrial dysfunction, and apoptosis, the use of rasagiline in PD is pathogenetically determined (Dias et al., 2013). It has been researched to have a powerful neuroprotective function: regulation of the mitochondrial apoptosis system, maintenance of the mitochondrial function, and increased expression of antioxidant enzyme genes (Naoi et al., 2020). The management of PD is relatively easy at the initial stages of the disease, where all dopamine-mimetic dopamine and drugs and amantadine or selegiline (or an antimuscarinic agent if the tremor is the main problem) can be very productive (Birkmayer and Riederer, 2012). As the disease progresses and these agents become insufficient, levodopa can be added (DeMaagd and Philip, 2015a). They compensate for the primary deficiency in PD and the decreased dopamine levels in the brain (Wu and Hallett, 2013).

## 6 Repurposed drugs that have been tested in clinical trials in PD

**FIGURE 2:**
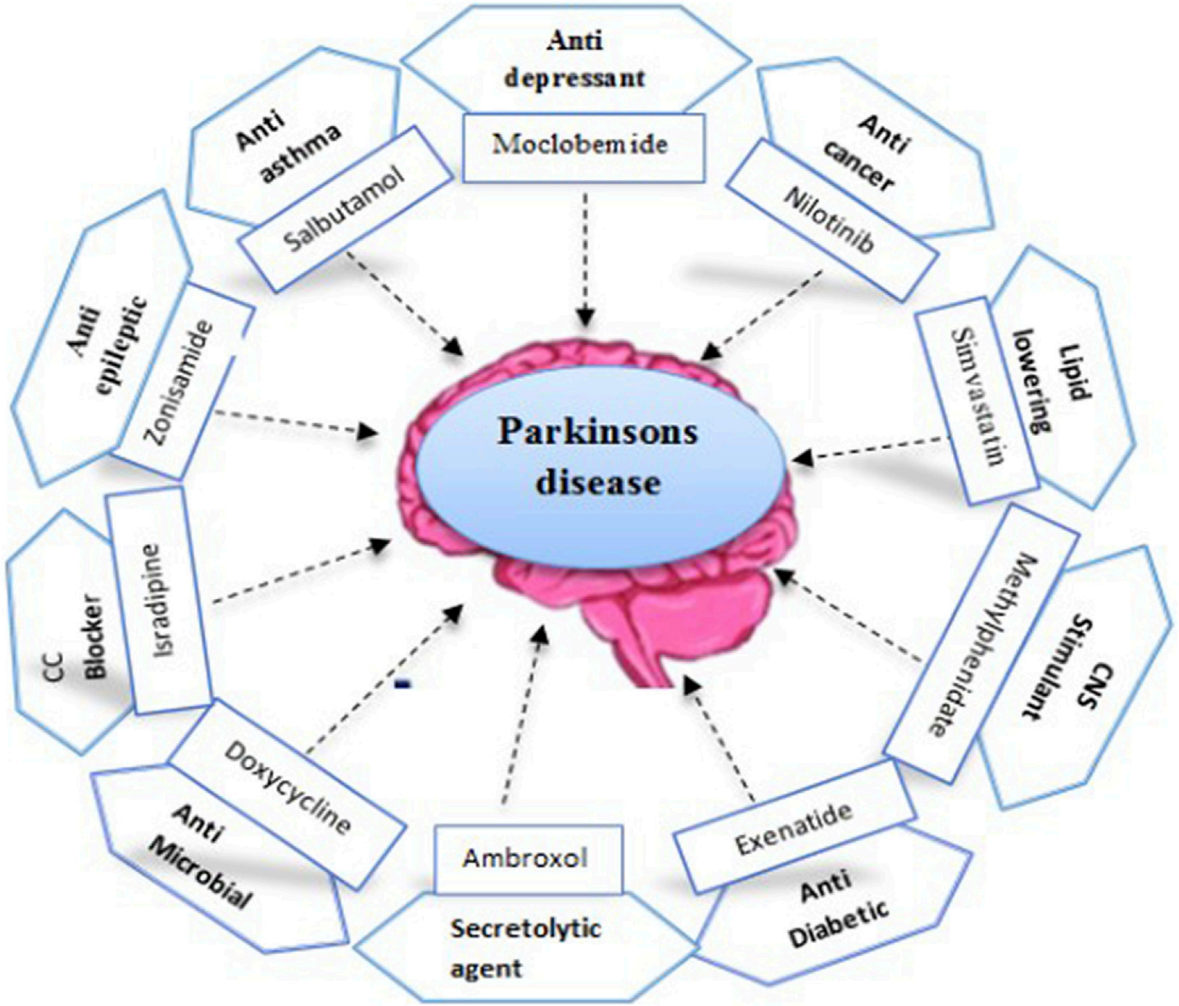
Repurposed drugs tested in clinical trials for Parkinson’s disease.

PD is a complicated disease, and until now, there are no disease-modifying treatments for PD (Warren Olanow and Kieburtz, 2010). Supportive therapies exist, like physiotherapy, medication (dopamine), and surgery, in rare cases, but still, there is a need for safer and more effective pharmacological treatments for psychosis in PD (Maiti et al., 2017). Figure 2 illustrates a complete list of drugs tested in clinical trials.

## 7 Conclusion

PD is a progressive concern in our society. There are, however, still numerous obstacles on the way to discovering methods for a cure. This literature review intended to give an overview of PD repositioned drugs that are currently in clinical trials and approved and are in use for PD. The aforementioned tables have comprehensive descriptions, including MOA and original, repurposed indications for each drug. We have demonstrated drugs that have already been repurposed and are suitable for PD, emphasizing the importance of finding disease-modifying therapies for PD. New drugs that have, in their pharmacodynamic effects, directed at the components of the pathogenesis of PD can be successfully studied as such therapy.
